# Automation of RNA-Seq Sample Preparation and Miniaturized Parallel Bioreactors Enable High-Throughput Differential Gene Expression Studies

**DOI:** 10.3390/microorganisms13040849

**Published:** 2025-04-08

**Authors:** Karlis Blums, Josha Herzog, Jonathan Costa, Lara Quirico, Jonas Turber, Dirk Weuster-Botz

**Affiliations:** Biochemical Engineering, TUM School of Engineering and Design, Technical University of Munich, Boltzmannstraße 15, 85748 Garching, Germany; karlis.blums@tum.de (K.B.); josha.herzog@tum.de (J.H.); j.costa@tum.de (J.C.); jonas.turber@tum.de (J.T.)

**Keywords:** parallel stirred-tank bioreactors, RNA-Seq, nanopore, automation, high-throughput, gene expression, *Saccharomyces cerevisiae*

## Abstract

A powerful strategy to accelerate bioprocess development is to complement parallel bioreactor systems with an automated approach, often achieved using liquid handling stations. The benefit of such high-throughput experiments is determined by the employed monitoring procedures. To gain a molecular understanding of the microbial production strains in miniaturized parallel single-use bioreactors, we extended the at-line monitoring procedures to transcriptome analysis in a parallel approach using RNA-Seq. To perform automated RNA-Seq experiments, we developed a sample preparation workflow consisting of at-line cell disruption by enzymatic cell lysis, total RNA extraction, nucleic acid concentration normalization, and Nanopore cDNA Library preparation. The pH-controlled aerobic batch growth of *Saccharomyces cerevisiae* was studied with six different carbon sources (glucose, pyruvate, fructose, galactose, sucrose, and mannose) on a 11 mL scale using 24 parallel stirred tank bioreactors integrated into a liquid handling station while performing at-line sample preparation for RNA-Seq on the same deck. With four biological replicates per condition, 24 cDNA libraries were prepared over 11.5 h. Off-line Nanopore sequencing yielded 20.97 M classified reads with a Q-score > 9. Differential gene expression analysis revealed significant differences in transcriptomic profiles when comparing growth with glucose (exponential growth) to growth with pyruvate (stress conditions), allowing identification of 674 downregulated and 709 upregulated genes. Insignificant changes in gene expression patterns were measured when comparing growth with glucose and fructose, yielding only 64 differentially expressed genes. The expected differences in cellular responses identified in this study show a promising approach for transcriptomic profiling of bioreactor cultures, providing valuable insights on a molecular level at-line in a high-throughput fashion.

## 1. Introduction

Key aspects of bioprocess design are the identification of optimal cultivation conditions for production strains to perform efficient fermentation processes. To accelerate these strategies, parallel bioreactor systems are complemented by automated and miniaturized approaches, often achieved by integrating parallel bioreactor systems into liquid handling stations (LHS) [1,2]. Advances in the design of miniaturized parallel stirred-tank bioreactors, particularly concerning oxygen input through the gas phase and control of all critical process variables, have contributed to scalable results, enabling high-throughput fermentations with at-line monitoring procedures [3,4,5,6]. Recently, individual online measurements of pH and dissolved oxygen concentrations, as well as at-line or online measurements of turbidity (as a measure of biomass concentrations), substrate, and metabolite concentrations using liquid handling stations, have been employed [6,7,8,9].

To gain a molecular understanding of the microbial production strains in miniaturized parallel bioreactors, we extended the at-line monitoring procedures to transcriptome analysis in a parallel approach using a next-generation sequencing approach via RNA-Seq [10]. Next-generation sequencing technologies have become standard procedure for genomic and transcriptomic studies [11,12]. RNA-Seq enables the identification and quantification of individual transcripts, which can be used to describe gene expression and, thus, the cellular response of the biological sample studied [13]. RNA-Seq can be performed using different technologies, such as common short-read technologies or long-read technologies developed by Oxford Nanopore Technologies (ONT) or Pacific Bioscience (PacBio) [14]. With the launch of ONT’s MinION, the availability of long-read next-generation sequencing (NGS) technology has significantly increased [15], and the usefulness of nanopore sequencing for studying transcriptional features in prokaryotic and eukaryotic microorganisms has been demonstrated [16,17,18].

**Figure 1:**
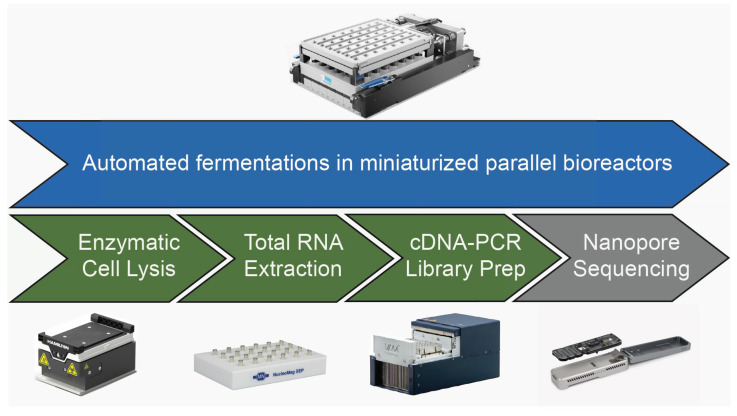
Overview of automated high-throughput operation of parallel bioreactors combined with at-line RNA-Seq sample preparation. The workflow steps of automated fermentation (shown in blue) and RNA-Seq sample preparation (enzymatic cell lysis, total RNA extraction, cDNA-PCR Library Prep, shown in green) were conducted in parallel by the liquid handling station, excluding centrifugation during enzymatic cell lysis. The pooling of samples and loading of the sequencing flow cell (Nanopore sequencing, marked in gray) was not automated and, thus, was performed manually.

Combining miniaturized parallel bioreactors with a high-throughput RNA-Seq sample preparation approach will allow a comprehensive comparison of microbial gene expression patterns in different production strains under various reaction conditions. To the best of our knowledge, such an automated, high-throughput, bulk RNA-Seq setup has not been published. The RNA-Seq sample preparation takes place on the same LHS where parallel fermentations occur. The automated at-line RNA-Seq sample preparation workflow described in this work (Figure 1) consists of at-line cell disruption by enzymatic cell lysis, followed by total RNA extraction and Nanopore cDNA library preparation. We chose to work with the nanopore sequencing technology since the compactness of the MinION makes it attractive for automated handling, thus, enabling a complete high-throughput RNA-Seq workflow on a single liquid handling device. This work was conducted using cDNA-PCR technology on multiplex samples on a single sequencing device, as the direct RNA sequencing technology did not have a multiplexing option available at the time of this study.

We opted to work with *Saccharomyces cerevisiae* to demonstrate the usefulness of the workflow and its high-throughput nature. *S. cerevisiae* is a well-studied eukaryotic microorganism and is widely used in a variety of biotechnological applications, ranging from industrial ethanol production to food biotechnology [19,20] and recombinant protein production [21,22]. Furthermore, metabolic engineering has additionally enabled the use of *S. cerevisiae* as a microbial cell factory, allowing the production of valuable chemical compounds, such as organic acids, sugar alcohols, furans, and glycerin [23,24,25]. The industrial relevance of *S. cerevisiae* in the biotechnological production of glutathione and isoprenoids has also been described [26,27]. The diversity of carbon sources utilized by *S. cerevisiae* contributes to the extensive range of biotechnological applications of this yeast, making carbon source utilization an important area of study in *S. cerevisiae*. We chose six different carbon sources to capture the differences in cellular responses of *S. cerevisiae* using our automated approach, including the preferred carbon sources, glucose and fructose [28]. We also chose the sugars galactose, mannose, and sucrose to compare the gene expression to the preferred carbon source. Galactose catabolism requires the involvement of genes of the Leloir pathway [29]. The hexose mannose does not induce catabolite repression compared to glucose and fructose [30], whereas the disaccharide sucrose requires hydrolysis as a first utilization step [31]. Finally, pyruvate was employed as a non-fermentable substrate to capture a gluconeogenic response.

## 2. Materials and Methods

### 2.1. Strain

A wild-type yeast strain *Saccharomyces cerevisiae* Meyen ex E.C. Hansen (DSM No. 1333) was used in this study. The strain was purchased from the German Collection of Microorganisms and Cell Cultures. The yeast cells were stored as cryo-cultures at −80 °C in 25% glycerol (*v*/*v*).

### 2.2. Media

The seed cultures were grown in a yeast extract peptone dextrose medium (YPD, 20 g L^−1^ yeast extract, 10 g L^−1^ peptone, 20 g L^−1^ glucose). The seed cultures were prepared in 1 L shake flasks without baffles, inoculated with 500 µL of the cryo-culture in 100 mL YPD Medium, and shaken overnight with 250 rpm at 30 °C. (Multitron, Infors HT, Bottmingen, Switzerland).

The composition of the defined medium for the parallel stirred-tank bioreactor cultivations was as follows: 40 g L^−1^ carbon source (glucose, pyruvate, galactose, mannose, fructose, or sucrose), 1 g L^−1^ MgSO_4_ · 7 H_2_O, 10 g L^−1^ (NH_4_)_2_SO_4_, 15 g L^−1^ KH_2_PO_4_, 100 mg L^−1^ tetracycline, 100 mg L^−1^ kanamycin, 1% (*v*/*v*) antifoam (Antifoam 204, Sigma-Aldrich, Taufkirchen, Germany), 27.2 mg L^−1^ CaCl_2_, 7.5 mg L^−1^ ZnSO_4_ · 7 H_2_O, 1.3 mg L^−1^ CuSO_4_ · 5 H_2_O, 1.3 mg L^−1^ CoCl_2_ · 6 H_2_O, 5.0 mg L^−1^ MnSO_4_ · H_2_O, 6.1 mg L^−1^ (NH_4_)_6_Mo_7_O_24_ · 4 H_2_O, 125 mg L^−1^ myo-inositol, 5 mg L^−1^ nicotinic acid, 6.78 mg L^−1^ calcium pantothenate, 5.6 mg L^−1^ thiamine hydrochloride, 7.53 mg L^−1^ pyridoxine hydrochloride, and 0.125 mg L^−1^ biotin. The kanamycin sulfate was dissolved in deionized water. The tetracycline hydrochloride was dissolved in 50% (*v*/*v*) ethanol in water mixture. The medium was supplemented with antibiotics as a precautionary measure to avoid contamination of the slower-growing yeast with bacteria. The titration agents were stored on the deck and were not sterile, as described further. A solution containing (NH_4_)_2_SO_4_, KH_2_PO_4_, antifoam, the carbon sources, and MgSO_4_ · 7 H_2_O were autoclaved separately at 121 °C for 20 min. The trace element and vitamin solutions and the antibiotics solutions were filter-sterilized (0.22 µL filter, Steritop, Merck, Darmstadt, Germany) and added to the medium aseptically before inoculation.

### 2.3. Parallel Stirred-Tank Bioreactors

**Figure 2:**
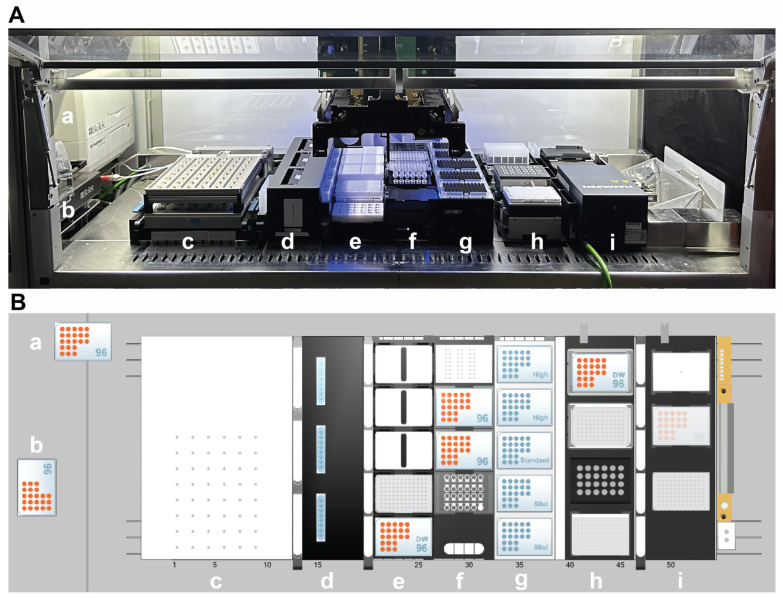
(**A**). Experimental setup for performing automated high-throughput fermentations with at-line RNA-Seq sample preparation. From left to right, the key labware used in the workflow consists of a microplate reader (a), a microplate washer (b), a parallel stirred-tank bioreactor system bioREACTOR48 DS mounted on the docking station (c), a pipetting needle washing station (d), containers for reagents and magnetic stands (e), microtiter plates and tubes for reagents (f), filtered tips (g), heater-shaker and cooling modules (h), and an on-deck thermocycler (i). (**B**). Top-down view of the deck layout as configured in the Venus software (Version 4.6, Hamilton Bonaduz AG, Bonaduz, Switzerland). The labels describe the same labware elements as in (**A**).

Batch fermentations were carried out on the mL scale employing a high-throughput bioreactor unit operating 24 stirred-tank bioreactors with gas-inducing stirrers (bioREACTOR48 DS, 2mag AG, Munich, Germany). Sterile single-use bioreactors with baffles (HTBD, 2mag AG, Munich, Germany) equipped with fluorometric sensors for online dissolved oxygen (DO) and pH measurements (PSt3-HG sensor for DO, LG1 sensor for pH, PreSens GmbH, Regensburg, Germany) were used with a working volume of 11 mL. The bioreactor unit was integrated into a liquid handling system (Microlab STAR, Hamilton Bonaduz AG, Bonaduz, Switzerland) equipped with 8 × 1000 µL pipetting channels, a needle washing station (Chemical Resistant Wash Station, Hamilton Bonaduz AG, Bonaduz, Switzerland), a plate handler (iSWAP, Hamilton Bonaduz AG, Bonaduz, Switzerland), a microplate washer (405 LS, Biotek, Winooski, VT, USA), and a microplate reader (Synergy H1, Agilent Technologies, Inc., Santa Clara, CA, USA), as shown in Figure 2.

For the automation of cell disruption, total RNA extraction, and cDNA Library Prep, the liquid handler was additionally equipped with heating and shaking modules (Hamilton Heater Shaker—HHS, Hamilton Bonaduz AG, Bonaduz, Switzerland) and cooling modules (Cold Plate Air Cooled Heater/Cooler—CPAC, Inheco Heating & Cooling GmbH, Martinsried, Germany), and an on-deck thermocycler (On Deck Thermal Cycler—ODTC, Inheco Heating & Cooling GmbH, Martinsried, Germany).

The lid of the bioreactor unit with mounted stirrers was autoclaved at 121 °C for 20 min. The sterile, single-use bioreactors were inserted into the bioreactor unit under a laminar flow hood. Before inoculation, the seed cultures were washed with phosphate-buffered-saline (PBS, 8 g L^−1^ NaCl, 0.2 g L^−1^ KCl, 1.44 g L^−1^ Na_2_HPO_4_, 0.24 g L^−1^ KH_2_PO_4_). After mixing the seed cultures with the media, 24 reactors were filled with the inoculated media (working volume of 11 mL). Next, the sterilized lid was placed on the bioreactor unit. The closed bioreactor unit was transported to the docking station (Docking Station for 2mag bioREACTOR48, Hamilton Bonaduz AG, Bonaduz, Switzerland) of the LHS (Figure 2), where all thermostat tubing connections are coupled in a single step during the docking process.

The temperature of the bioreactor unit was controlled at 30 °C (Dyneo DD-300F, Julabo GmbH, Seelbach, Germany), and the headspace was cooled to 20 °C (DLK 402, FRYKA-Kältetechnik GmbH, Esslingen, Germany). The headspace of each reactor was rinsed with 0.1 L min^−1^ sterile, moisturized air. The gas-inducing stirrers [3,32] were operated at 3000 rpm. Six fluorometric readers (MCR v5, PreSens GmbH, Regensburg, Germany) were used to perform parallel measurements of the pH and DO of each reactor every 40 s.

Steel needles with a maximum pipetting volume of 300 µL were used for sampling and pH control. After each liquid handling step, the needles were disinfected with 70% (*v*/*v*) ethanol and washed in the needle-washing station with sterile, deionized water. Sampling of 150 µL was carried out every hour for the first seven hours and every two hours afterward. An additional sampling was carried out at 6.5 h. Samples for optical density (OD_600_) measurements were collected in a microtiter plate (MTP) and diluted 10-fold and 100-fold with PBS in a second MTP. The MTP containing the diluted samples was transported to the microplate reader. Both MTPs were washed in the microplate washer after each OD_600_ measurement. Based on the online pH measurements, pH control was conducted using proportional control. A total of 2 M H_3_PO_4_ and 12.5% (*v*/*v*) NH_4_OH were prepared in 400 mL containers and stored on deck. A volume ranging from 10 to 45 µL of the acid or base was added to the reactors based on the deviation from the pH setpoint of pH 6.0. A dynamic priority-based scheduling software (demo version provided by 2mag AG (Munich, Germany) and Hamilton Bonaduz AG (Bonaduz, Switzerland) for testing purposes) was used to assign and execute pH control and sampling tasks [33]. The sampling task was split into four steps, where after each step, the priority of the sampling task was reset to the base priority, allowing execution of pH or RNA-Seq sample preparation in between.

### 2.4. Automated RNA-Seq Sample Preparation Workflow

An automated RNA-Seq sample preparation workflow for 24 reactors was developed and optimized with the LHS (Hamilton STAR, Hamilton Bonaduz AG, Bonaduz, Switzerland), which included enzymatic cell lysis, total RNA extraction, nucleic acid concentration normalization, and cDNA library prep. Each protocol was validated manually before proceeding with automation. The at-line RNA-Seq sample preparation workflow was triggered based on the OD_600_ signal. Once the mean OD_600_ of the stirred-tank reactors operated with glucose exceeded 2.5, sampling was conducted for all reactors. The at-line RNA-Seq sample preparation workflow was performed parallel to the fermentation, where the steps of RNA-Seq sample preparation were permanently assigned the highest priority. In between RNA-Seq sample preparation steps, the dynamic priority-based calculation for pH control and sampling was applied. A liquid class for each pipetted reagent was developed, and the trueness and precision of the liquid classes are shown in Supplementary Table S2 and Table S3. The dispensed volume was estimated using a gravimetric measurement by weighing the target tube (PCR Tube 0.2 mL, Eppendorf, Hamburg, Germany) pre- and post-dispense. The trueness and precision (coefficient of variation, CV) were calculated as follows:

Trueness = ((V_M_ − V_T_) × V_T_^−1^) × 100% (1)

CV = (SD × V_M_^−1^) × 100% (2)

with V_M_ being the mean transferred volume, V_T_ being the target volume, and SD the standard deviation.

The samples were collected in deep-well plates (DWP, Macherey-Nagel GmbH, Düren, Germany) for enzymatic cell lysis. A correlation of OD_600_ and cell count was used to calculate the volume to sample 2 × 10^7^ cells per reactor (the sampling volume is shown in Supplementary Table S1). The DWP containing the samples was centrifuged off-line for 10 min at 5000× *g* (Hettich, Tuttlingen, Germany). Since deck space was limited, an on-deck centrifuge was not integrated, meaning centrifugation during enzymatic cell lysis was carried out manually, where the deep-well plate containing the samples was removed from the LHS. During this time, sampling and pH control tasks were continued normally. During transport to the centrifuge, the plate was covered with a sealing lid (Adhesive sealing films, VWR, PA, USA). After centrifugation, the plate was returned to the LHS. The sampling for RNA extraction was conducted using the 300 µL steel needles. In contrast, all steps in the RNA-Seq sample preparation workflow were performed using filtered, conductive 50 µL, 300 µL, or 1000 µL Hamilton CO-RE II Tips (Hamilton Bonaduz AG, Bonaduz, Switzerland). After discarding the supernatant, the samples were incubated in 600 µL sorbitol buffer (1 M sorbitol, 100 mM EDTA (pH 8.0), 14 mM 2-Mercaptoethanol) and 100 U of lyticase (Lyticase from *Arthrobacter luteus*, Sigma-Aldrich, Taufkirchen, Germany) for 30 min at 30 °C on the HHS. After incubation, the DWP was removed from the LHS to pellet the spheroblasts by centrifugation for 10 min at 300× *g*. The DWP was returned to the LHS, and after removing the supernatant, automated, magnetic bead-based total RNA extraction was performed using the Mag-Bind Total RNA 96 Kit (Omega Bio-Tek, Inc., Norcross, GA, USA). In brief, the proteins and DNAse are degraded using Proteinase K and DNAse, and the total RNA is bound on magnetic beads and eluted afterward. Reagents were prepared in 1.5 mL and 2 mL tubes (RNAse-free Microcentrifuge Tubes, Thermo Fischer Scientific Baltics, Vilnius, Lithuania), as well as 60 mL trays (60 mL reagent reservoirs, Hamilton Bonaduz AG, Bonaduz, Switzerland). For each reagent placed on the deck, a dead volume of at least 10% was accounted for to enable more precise pipetting. The samples were eluted in a final volume of 65 µL. A rod-based magnetic stand (NucleoMag SEP, Macherey-Nagel GmbH, Düren, Germany) was used for magnetic separations during RNA extraction.

To normalize the input amount for the cDNA Library Prep, the RNA was quantified by an automated absorbance measurement using the microplate reader on a microvolume plate (Take3 Trio, Agilent Technologies, Inc., Santa Clara, CA, USA). A custom lid was designed to enable automated handling of the microvolume plate (Supplementary Figure S6). For the automated quantification of nucleic acid concentration, 5 µL of the extracted RNA of each sample was dispensed on the base of the Take3 Trio plate. The plate handler was used to transport the custom lid to the base of the Take3 Trio plate, which was subsequently transported to the microplate reader by the plate handler, where absorbance was measured at 260 nm. An off-line absorbance measurement of the RNA samples using a nanophotometer (NanoPhotometer N120, Implen Gmbh, Munich, Germany) was conducted to confirm the purity (Supplementary Table S1).

The cDNA libraries for nanopore sequencing were prepared by automation of the SQK-PCB114.24 kit (Oxford Nanopore Technologies, Oxford, UK). Briefly, the polyadenylated RNA is reverse transcribed to cDNA and amplified via PCR using the on-deck thermocycler. The input amount of total RNA was increased to 1000 ng. The workflow includes two automated clean-up steps using magnetic beads. Reagents were prepared in a PCR plate (Hard-Shell^®^ 96-Well PCR Plate, Bio-Rad Laboratories GmbH, Feldkirchen, Germany) and 2 mL tubes, which were both cooled to 8 °C on the CPAC modules during the RNA-Seq sample preparation workflow. Mastermixes of reagents were prepared beforehand to increase the pipetting volume and, thus, pipetting trueness and precision (Supplementary Table S2). An excess of 14 µL was prepared for all reagents in the PCR plate (excluding barcode primers). To further accommodate pipetting of low volumes, after washing with a short fragment buffer, the sample volume was doubled by eluting in 24 µL. The volume of reagents was also doubled until final elution in 15 µL. The cDNA was amplified by PCR over 16 cycles. A ring-based magnetic stand (Levitation G-96PLT, Ariumlab, Tallinn, Estonia) was used for magnetic separation during cDNA Library Prep.

After eluting the 24 cDNA libraries, the samples were collected and handled manually. Furthermore, the pipetting tips were manually refilled twice during sample preparation. The cDNA concentration was quantified using a fluorescence-based assay (Quant-IT dsDNA Broad-Range, Invitrogen, OR, USA). An additional AMPure XP bead (Beckman Coulter, Brea, CA, USA) cleanup step was performed to pool 100 ng of each library, and 75 ng of the pooled library was loaded onto the flow cell.

### 2.5. RNA-Seq Data Acquisition and Analysis

Sequencing was performed over 72 h using a MinION Mk1B device (Oxford Nanopore Technologies, Oxford, UK) with an R10.4 flow cell. Basecalling was performed using Dorado (release 7.6.7, Oxford Nanopore Technologies, Oxford, UK) with the Super Accuracy model and a Q-score cutoff of Q9 (https://github.com/nanoporetech/dorado accessed on 6 November 2024). Pychopper (release 2.7.9, Oxford Nanopore Technologies, Oxford, UK) was used to identify, orient, and trim full-length cDNA reads (https://github.com/epi2me-labs/pychopper accessed on 6 November 2024). A reference *Saccharomyces cerevisiae* R64-1-1 transcriptome was generated by combining cDNA transcripts of genes and an ncRNA reference acquired from Ensembl [34]. The Pychopper-filtered reads were aligned to the reference transcriptome using minimap2 (with the following parameters: -ax map-ont -uf -p 1.0, release 2.28-r1209) [35,36]. Counts were generated using salmon quant (release 1.10.3) in the alignment-based mode without using an error model (--noErrorModel) [37]. Alignment statistics were acquired using samtools (release 1.10) [38] and seqkit (release 2.9.0) [39].

Protein-coding transcripts were summarized for gene-level analysis using tximport [40]. Differential gene expression analysis was performed using edgeR (release 4.0.16) [41]. Gene counts were filtered using the edgeR function filterByExpr with the default parameters. Differential genes were identified using a generalized linear model in combination with *t*-tests relative to a threshold (glmTreat from the edgeR package). The threshold for differential expression was set to a fold-change of 1.3, and the Benjamini–Hochberg adjusted *p*-value to 0.01 [42]. The choice for the fold-change threshold was based on the recommendations of the edgeR authors when using glmTreat, which provides a formal approach for combining statistical significance and fold-change to identify biologically relevant genes [43]. Gene set enrichment analysis was performed using clusterProfiler based on gene ontology terms [44].

### 2.6. Data Availability

Sequencing data have been deposited in the Sequence Read Archive (SRA) in a fastq format with the accession number PRJNA1231053.

## 3. Results

### 3.1. Batch Growth of Saccharomyces cerevisiae Utilizing Different Carbon Sources

**Figure 3:**
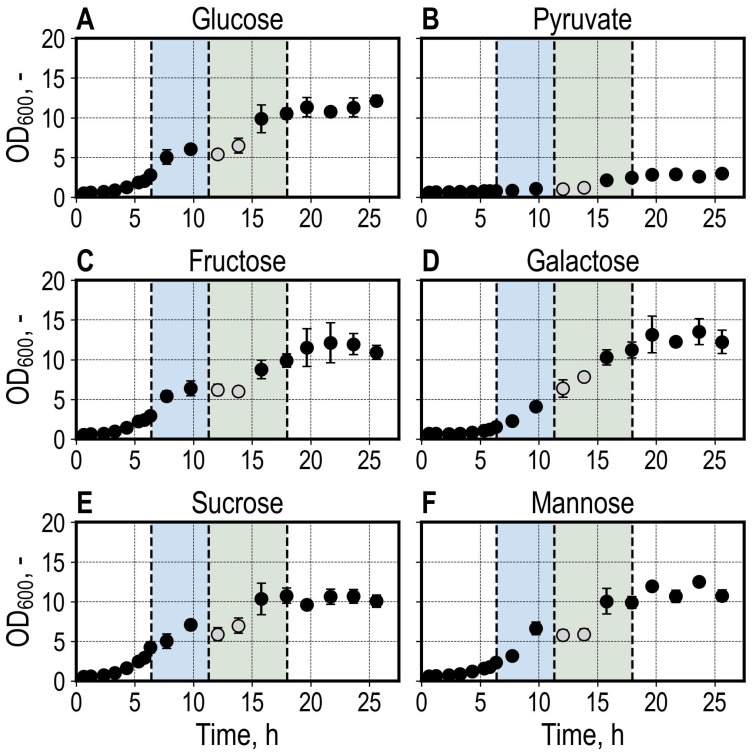
Growth of *S. cerevisiae* based on optical density (OD_600_) during batch cultivation (40 g L^−1^ carbon source) in the bioreactor unit (bioREACTOR48 DS) with four biological replicates per condition. Growth with glucose (**A**), pyruvate (**B**), fructose (**C**), galactose (**D**). sucrose (**E**), mannose (**F**). OD_600_ measurements were carried out by the LHS every hour until 7 h and every two hours afterward. The enzymatic cell lysis and total RNA extraction, depicted by the blue area, was started for all conditions at t = 6.4 h, where the mean OD_600_ of the glucose condition had reached OD_600_ > 2.5, and finished at t = 11.3 h. Library Prep was started immediately after and finished at t = 17.9 h, as shown by the green area. Samples shown in gray at t = 12.1 h and t = 13.8 h were stored in the microtiter plate for longer than 10 min due to an ongoing Library Prep task, and the measurement was possibly affected by sedimentation. The following process parameters were set for the parallel batch cultivations: V = 11 mL, gas flow rate = 0.1 L min^−1^, stirrer speed = 3000 rpm, and T = 30 °C, pH = 6.0.

After a lag phase of approximately 3 h, *S. cerevisiae* cultures growing with glucose, fructose, and sucrose showed exponential growth before switching to growth with the produced ethanol after consuming the primary carbon source (Figure 3). The glucose, fructose, and sucrose cultures exhibited similar growth characteristics. However, the sucrose cultures had reached a higher OD_600_ at the time of sampling (Figure 3E). The samples diluted for OD_600_ measurements at t = 12.1 h and t = 13.8 h were exposed to sedimentation effects (11 min, and 15 min, respectively, otherwise 5 min on average), as the LHS switched to carrying out a cDNA Library Preparation step after diluting the samples. The cultures grown with galactose (Figure 3D and Supplementary Figure S1D) and mannose (Figure 3F and Supplementary Figure S1F) showed a more prolonged lag phase and slower growth, reaching a similar final OD_600_ of 10–12 compared to the other conditions. Growth with pyruvate was limited, reaching a final OD_600_ of 3 (Figure 3B).

**Figure 4:**
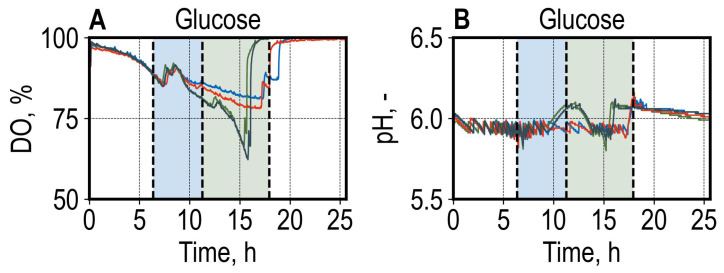
Dissolved oxygen (DO) (**A**) and pH (**B**) on-line measurements of *S.cerevisiae* cultures grown with glucose during batch cultivation in the bioreactor unit (bioREACTOR48 DS) with four biological replicates per condition. Each colored line represents a biological replicate. The enzymatic cell lysis and total RNA extraction, depicted by the blue area, were started at t = 6.4 h, where the mean optical density (OD_600_) of the glucose condition had reached OD_600_ > 2.5, and finished at t = 11.3 h. Library Prep was started immediately after and finished at t = 17.9 h, as shown by the green area. The following process parameters were set for the parallel batch cultivations: V = 11 mL, gas flow rate = 0.1 L min^−1^, stirrer speed = 3000 rpm, T = 30 °C, pH = 6.0.

Reproducible DO courses were observed between biological replicates during the initial batch phase. After finishing consumption of the provided carbon source, the cultures showed different DO usage patterns (shown for cultures grown with glucose in Figure 4A; the course of DO and pH for all conditions are available in Supplementary Figure S1). This was also observed in the pH measurements, where the course of pH differed after the primary carbon source was consumed for cultures of the same condition (Figure 4B). It could be shown that the differences in the growth patterns illustrated by the DO and pH signals are based on different patterns of acetate consumption, which was produced during the initial batch phase (Supplementary Figure S3). The differences in acetate consumption were not investigated further.

All reactors were sampled for total RNA extraction at t = 6.4 h once the glucose reactors reached an OD_600_ mean of >2.5 (Figure 3). At this stage, cultures growing with glucose, fructose, and sucrose were reaching the end of the exponential growth phase. In contrast, the cultures growing with galactose and mannose were sampled at the initial stages of the exponential growth phase, whereas cultures growing with pyruvate showed minimal growth during sampling for total RNA extraction.

The total volume sampled for OD_600_ measurements was 2700 µL within 26 h. On average, 134 µL was sampled for RNA Extraction (Supplementary Table S1). Considering an average volume of 527 µL added in the form of titration agents, an average final volume of 24.6% was withdrawn. Although the volume withdrawn seems to be high, the scalability of the parallel bioreactor system employed in this work has been shown for yeast from 10 mL up to 1000 L [5].

### 3.2. Automated High-Throughput RNA-Seq Sample Preparation

The RNA-Seq analysis workflow for 24 samples was carried out over 11.5 h (5 h RNA extraction, 6.5 h cDNA library prep) while performing parallel fermentations (Figure 3). Off-line centrifugation was carried out at 6.52 h, 7.49 h, and 7.98 h, whereas tips were refilled at 10.74 h and 16.38 h. During this time, the control of bioreactors continued normally.

**Figure 5:**
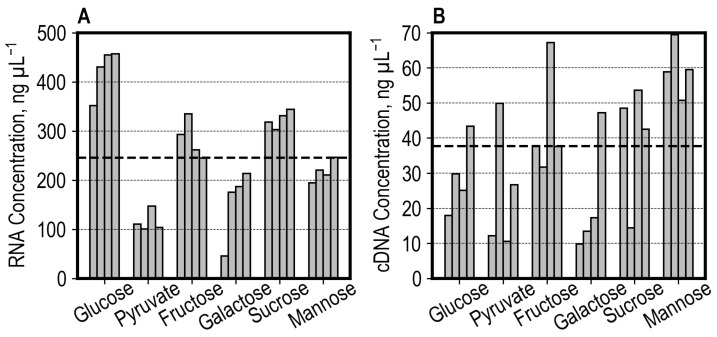
Measured RNA (**A**) and cDNA (**B**) concentrations from samples harvested from *S. cerevisiae* cells utilizing glucose, pyruvate, fructose, galactose, sucrose, and mannose. Each bar represents a biological replication. The dashed horizontal lines represent the median value (median of RNA concentration 245.8 ng µL^−1^; median of cDNA concentration 37.7 ng µL^−1^). The total RNA concentration was measured based on absorbance by the LHS using the Take3 Trio plate (**A**). The cDNA concentration was measured manually using a fluorescence-based hybridization assay (**B**).

After extracting the total RNA, a median RNA yield of 245.8 ng µL^−1^ was measured among all 24 samples, with the median RNA yield of 107.2 ng µL^−1^ for the cultures grown with pyruvate being significantly lower (Figure 5). The median RNA yield of 442.3 ng µL^−1^ for cultures grown with glucose notably exceeded the overall median. One sample of the galactose condition was not successfully dispensed on the Take3 Trio plate and, thus, showed a distorted measurement. As the at-line RNA concentration measurements were used for normalizing the RNA input for the cDNA Library prep, underestimating the RNA yield in the galactose sample contributed to using twice as much input RNA (for this sample). The absorbance was additionally measured off-line for reference and to confirm the RNA quality by absorbance ratios (Supplementary Table S1).

A median cDNA yield of 37.7 ng µL^−1^ over 24 cDNA libraries was measured. In contrast to the extracted RNA yield, the cDNA yields showed a higher degree of scattering among biological replicates of the same condition, as shown in Figure 5. Contributing factors are most likely the pipetting of critical low-volume reagents (such as the reverse transcriptase and PCR primer solutions), where the trueness and CV were >10% when pipetting reagents of volumes as low as 2 µL with standard 1000 µL channels, as shown in Supplementary Table S2.

A total of 100 ng of each library was pooled for off-line sequencing, except for the second sample of the sucrose condition, where only 3 µL was available after final elution, meaning only 43.2 ng was pooled.

### 3.3. Off-Line Nanopore Sequencing

**Figure 6:**
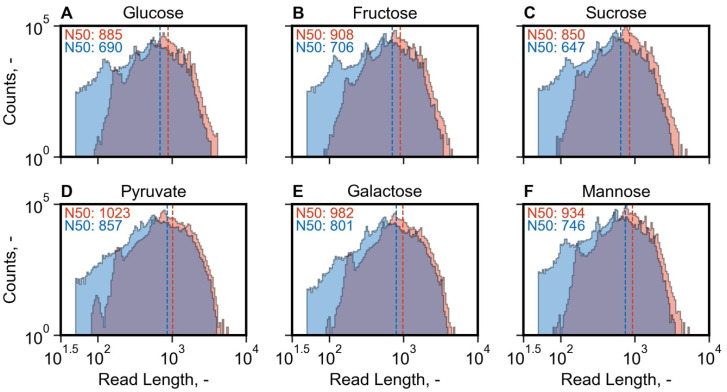
Read length distributions for selected libraries of each condition (growth with glucose (**A**), pyruvate (**B**), fructose (**C**), galactose (**D**). sucrose (**E**), mannose (**F**)). The raw read distributions (Q-score > 9) are shown in red, whereas the trimmed, full-length read distributions identified by Pychopper are in blue. Dashed vertical lines show the N50 values in bp corresponding to the color of each distribution.

We sequenced 24.4 M reads over 72 h, of which 21.73 M had a Q-score > 9 (read quality vs. read length is shown in Supplementary Figure S4). The overall N50 of all reads was 918 bp. The unclassified reads were discarded, and further analysis was undertaken with 20.97 M reads classified by barcoding primers. The read length distributions for a library of each condition are shown in Figure 6.

The effect of filtering for full-length reads and sequence trimming is reflected in the lower N50 values compared to raw N50 metrics (Figure 6). Differences between library sizes for different conditions among biological replicates did not reveal any correlation between library size and condition, as each condition had libraries showing a range of library sizes as described by the N50 values (Supplementary Figure S5).

**Figure 7:**
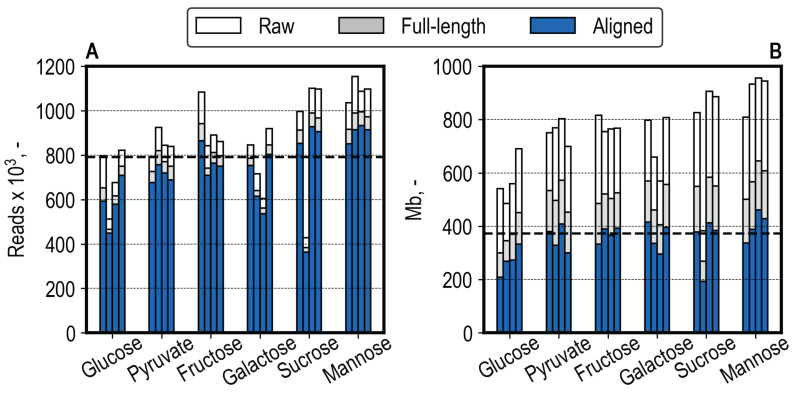
(**A**). Sequencing yield in reads. The white bars represent all reads with a Q-score of >9. The gray bars represent the share of reads identified as full-length after primer identification with Pychopper. The blue bars depict the proportion of reads aligned to the reference transcriptome using minimap2. (**B**). Sequencing yield in bases. The white bars represent all bases corresponding to all reads with a Q-score of >9. The gray bars represent the number of bases corresponding to full-length reads. The blue bars represent the bases corresponding to aligned reads, excluding clipping (based on CIGAR). The vertical black lines represent the median aligned values of 790.93 k reads and 372.05 Mb.

The read and base statistics in raw, full-length, and aligned metrics are shown in Figure 7 for each sample. A high percentage of reads were identified as full-length (on average 89.8%, ranging from 81.9% to 92.8%, Figure 7A). A mean of 93.9% of full-length reads were aligned to the reference transcriptome as primary alignments. In contrast to the read metrics, a lower average amount of 66.3% (range of 55.5% to 71.3%) bases corresponded to full-length reads when compared to the total bases sequenced for reads with a Q-score > 9, which is explained by the removal of long reads, which were not identified as full-length, and trimming during Pychopper filtering (Figure 7B). An average of 71.2% (range of 66.0% to 77.4%) of bases corresponding to full-length reads were aligned to the transcriptome (described by the CIGAR string, which excludes clipped regions), indicating clipping during alignment when comparing the high proportion of aligned reads to the aligned bases. The median aligned read count among all cDNA libraries was 790.93 k, amounting to 372.05 Mb aligned bases. A lower sequencing yield (192 Mb aligned bases) was measured for the sucrose library, where less than half of the desired cDNA amount was pooled due to insufficient cDNA available (Figure 5B). Additionally, the samples of the glucose condition showed a lower yield (Mb) than the median of the aligned bases (208 Mb to 333 Mb). As mentioned, the differences in the cDNA library (Figure 5B) and sequencing yields (Figure 7) between samples can be explained by the lower trueness and precision when pipetting low-volume reagents (Supplementary Table S2).

### 3.4. Differential Gene Expression Analysis

The reads were aligned to a reference transcriptome, which allows the identification and quantification of annotated transcripts included in the transcriptome but does not enable the identification of new transcripts [45]. This study aimed to identify differences between reaction conditions in parallel stirred-tank bioreactors on a molecular level, and since the chosen conditions should show expected cellular responses in the well-studied *Saccharomyces cerevisiae*, an alignment to a reference transcriptome was sufficient for this purpose.

**Figure 8:**
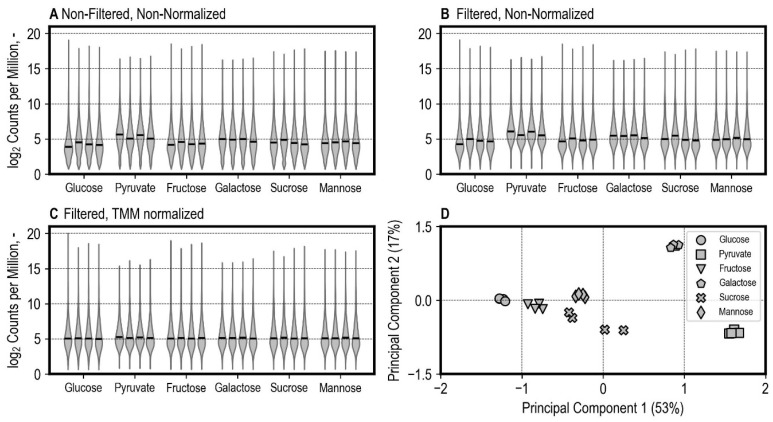
Gene expression distributions are described by log_2_ counts per million for each library. The horizontal black lines depict the median log_2_ counts per million. Distribution of non-filtered and non-normalized counts (**A**), filtered and non-normalized counts (**B**), and filtered and normalized counts using the trimmed mean of M values (TMM) method (**C**). The result of principal component analysis (**D**) describes the relationships between gene expression profiles between libraries.

On average, a fraction of 86% (range of 81–89%) protein-coding transcripts included in the reference transcriptome were identified with at least one count after salmon quantification. Following quantification, the protein-coding transcripts were summarized for gene-level analysis using tximport. The distributions of raw, filtered, and TMM (trimmed mean of M values [46]) normalized log_2_ counts per million are shown in Figure 8. Principal component analysis shows a high consistency of biological replicates after normalization, showing clear clusters for each condition (Figure 8D). A lower level of clustering was observed for the sucrose condition.

**Figure 9:**
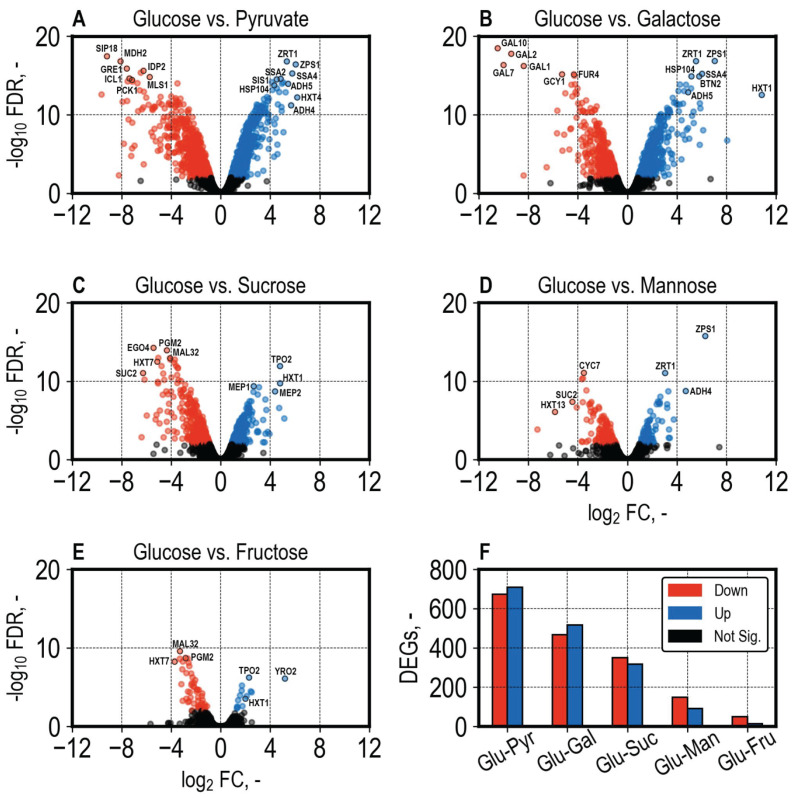
(**A**–**E**). Volcano plots showing the -log_10_ Benjamini–Hochberg adjusted *p*-values (False Discovery Rate, FDR) and log_2_ (Fold Change, FC) of all genes between pairwise condition comparisons. The pH-controlled aerobic growth of *S. cerevisiae* with pyruvate, galactose, sucrose, mannose, and fructose was compared to growth with glucose. The threshold for differentially expressed genes was set to an FDR > 0.01 and fold change of 1.3, as recommended by edgeR authors when using generalized linear models in combination with *t*-tests relative to a threshold (glmTreat from the edgeR package). (**F**). Amount of the identified down- and upregulated genes, illustrating the extent of differences in cellular responses between conditions. Genes identified as downregulated are marked in red and upregulated in blue. Non-significant genes that do not pass one of the thresholds are marked in black. The abbreviations are Glu: Glucose, Pyr: Pyruvate, Gal: Galactose, Suc: Sucrose, Man: Mannose, Fru: Fructose, and DEGs: Differentially expressed genes.

We compared the gene expression of all conditions to growth with glucose and identified apparent differences in cellular responses (Figure 9). The comparison of growth with glucose and pyruvate yielded 709 up- and 674 downregulated genes (Figure 9A), whereas the comparison of growth with glucose and fructose yielded only 64 differentially expressed genes (Figure 9E). The amounts of differentially expressed genes (DEG) between comparisons, as depicted in Figure 9F, correspond to expected cellular responses. The comparison of glucose and pyruvate yielded the most DEGs, as the cultures growing with glucose were in the exponential growth phase (Figure 3A and Figure 4A), whereas, in the pyruvate condition, the cultures were stationary (Figure 3B and Figure 3B and Figure S1B), corresponding to stress conditions. Similarly, the cultures growing with galactose during sampling were at the beginning of the growth phase (Figure 3D) and showed a high amount of DEGs compared to growth with glucose (Figure 9B). The least amount of DEGs was identified in the comparisons of glucose vs. mannose and glucose vs. fructose (Figure 9F). The galactose and mannose cultures were sampled in the early exponential growth phase, compared to the glucose condition, where the cultures were nearing the end of the exponential growth phase. This may have resulted in differences in physiological states, which can contribute to different gene expression profiles, in addition to the utilization of different carbon sources.

To confirm the viability of our workflow to capture the expected cellular responses, we first looked at the most significantly up- and downregulated genes for each condition. In the glucose and pyruvate comparison, the most overexpressed genes (among others) in pyruvate cultures were associated with gluconeogenesis (*PCK1*), glyoxylate cycle (*MLS1*, *MDH2*, *ICL1*), osmotic stress response (*SIP18*, *GRE1*), and non-fermentable carbon source metabolism (*IDP2*), consistent with previously published data on transcriptional regulation of nonfermentable carbon utilization in *S. cerevisiae* [47]. In contrast, genes involved in glycolysis (*ENO2*), ethanol production (*ADH4*, *ADH5*), ethanol stress response (*BTN2*, *SSA2*, *SSA4*, *SIS1*), and zinc uptake (*ZRT1*, *ZPS1*) were identified as overexpressed with glucose compared to pyruvate. The involvement of *BTN2* and *HSP70* family genes in ethanol stress response has been reported previously [48,49].

In the glucose and galactose comparison (Figure 9B), galactose metabolism (*GAL2*, *GAL10*, *GAL7*, *GAL1*), galactose-inducible (*GCY1*, *FUR4*), and sulfate metabolism (*MET3*, *MET8*) genes were upregulated in galactose cultures. Genes involved in sucrose metabolism (*SUC2*, *PGM2*, *MAL32*) were among the top upregulated genes for cultures growing with sucrose (Figure 9C).

The overexpression of genes involved in increased zinc uptake (*ZRT1* and *ZPS1*) and *ADH* family genes was consistent in the comparisons of late exponential growth cultures (glucose) with stationary (pyruvate) or early exponential growth phase cultures (galactose and mannose). The involvement of zinc in ADH protein catalytic activity, which reduces acetaldehyde to ethanol, has been shown previously [50,51].

**Figure 10:**
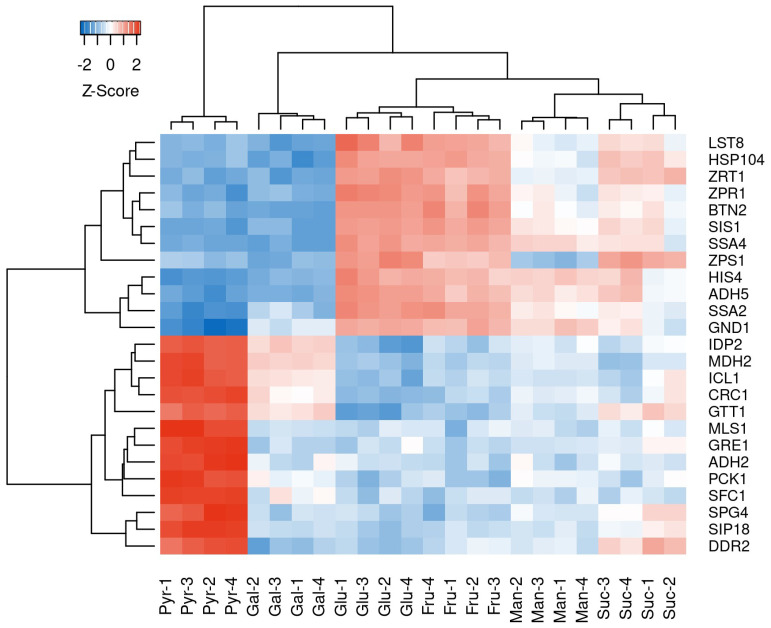
Clustered heatmap of the expression (z-score normalized log_2_ counts per million) of the top 25 differentially expressed genes identified in the glucose vs. pyruvate comparison. Expressions are shown for all libraries. The red color indicates a positive difference in expression from the mean, and the blue color depicts a negative difference.

We selected the top 25 differentially expressed genes in the glucose and pyruvate comparison and inspected the gene expression of all samples for these genes (Figure 10). The selected genes highlight the different states of the cultures during sampling for RNA extraction. The pyruvate and galactose cultures (stationary and early growth phase) clustered together and showed similar expression patterns of downregulation for the genes involved in ethanol stress response (*HSP104*, *BTN2*, *SIS1*, *SSA4*, *SSA2*). Conversely, the similarity in cellular response of the glucose and fructose cultures is illustrated by the highly comparable expression patterns of the mentioned genes.

**Figure 11:**
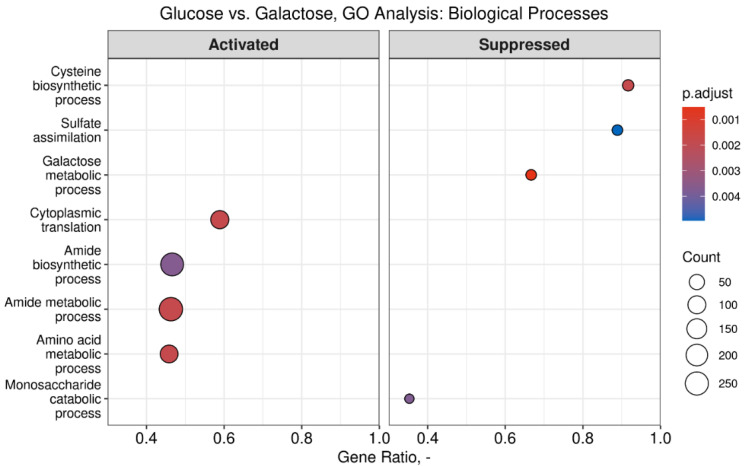
The result of gene set enrichment analysis shows top enriched biological processes based on gene ontology (GO) terms in the comparison of *S. cerevisiae* growth with glucose and galactose. The size of the dots depicts the size of the gene set describing the GO term. The gene ratio shows the proportion of differentially expressed genes in the gene set.

To confirm the functional profile of the cultures on a more comprehensive scale, the identified DEGs were subjected to gene set enrichment analysis using gene ontology, shown for the glucose and galactose comparison as an example (Figure 11). The top suppressed (compared to glucose) biological processes of cytoplasmic translation, amide biosynthetic and metabolic processes, and amino acid metabolic processes, underline the lower growth rate of the galactose cultures validated by lower protein biosynthesis activity. The activated metabolic galactose process corresponds well to the onset of the growth phase by galactose utilization. Additionally, we identified activated biological processes involved in sulfur metabolism in the form of cysteine biosynthetic process and sulfate metabolism in the galactose cultures (Figure 11).

## 4. Discussion

We established a high-throughput experimental approach for assessing bioreactor cultures on a molecular level using RNA-Seq. Using our automated workflow, we extracted total RNA and prepared cDNA libraries for 24 samples in 11.5 h using nanopore sequencing while operating 24 parallel bioreactors. Our data show the expected cellular responses of the cells corresponding to the growth phase during sampling and the utilization of the available carbon source in the medium.

Automation of genomic applications is becoming increasingly common [52,53,54] and has already been shown to heavily increase throughput in transcriptomic studies [55,56,57]. We believe the increased accessibility of sequencing technologies can accelerate bioprocess development and complement existing high-throughput strain screening and improvement strategies. One of the main advantages of our approach for high-throughput fermentation using automation of parallel bioreactors is the high level of scalable biological replication, which is essential in differential expression studies [58].

Our study aimed to identify overall differences in gene expression to demonstrate the potential of using the automated RNA-Seq sample preparation workflow for identifying potential targets for strain improvement in screening experiments using industrial production strains. We illustrated the expression patterns of the top up- and downregulated genes, which was consistent with the literature. A more comprehensive analysis of the RNA-Seq data presented in this work could yield further valuable insights into gene expression patterns of *S. cerevisiae* utilizing the carbon sources studied. To better capture differences between carbon source utilization, it would be more suitable to sample all the cultures at the same state in the exponential growth phase to ensure comparable physiological conditions. In our workflow, all reactors were sampled at the same process time, which resulted in comparisons of early exponential growth phase (for galactose and mannose cultures) and late exponential growth conditions (glucose cultures), contributing to different gene expression profiles not only due to the utilization of different carbon sources but also due to the different physiological state. When combining the operation of parallel bioreactors and RNA-Seq sample preparation, we consider the immediate processing of the RNA samples up to cDNA libraries an advantage, as delaying the processing of reactor culture samples would lead to changes in cellular processes. Delaying the inoculation or inoculating with a higher cell density would be viable options to capture the same physiological state among different conditions in a single parallel experiment for all reactors simultaneously.

The cDNA library and sequencing yields showed a degree of scattering (Figure 5B and Figure 7), which could be improved by further liquid class development for critical low-volume reagents. The CV and trueness of some 2 µL liquid transfers in our workflow exceeded 10% (Supplementary Table S2), which most likely had an impact on the consistent preparation of libraries. In our workflow, we used the same machine for performing high-throughput fermentations, enzymatic cell disruption, RNA extraction, and cDNA library preparation, which requires pipetting of a wide variety of solutions and reagents in different volumes, enabled by equipping the liquid handler with the standard 1000 µL channels. To pipet low volumes, such as 1 or 2 µL, pipetting channels based on acoustic or magnetic displacement would be more suitable [59,60].

Reliability and efficiency can be increased by dedicating a second liquid handler for at-line RNA-Seq sample preparation with specialized low-volume pipetting options. Additionally, deck space would be less limiting, allowing more space for tips and an on-deck centrifuge, thus, enabling a complete walk-away solution.

Currently, the RNA-Seq sample preparation workflow is applicable to polyadenylated RNA, as the SQK-PCB114.24 library protocol amplifies polyadenylated RNA. In vitro polyadenylation and rRNA depletion must be employed to extend the automated workflow to prokaryotic microorganisms, as shown for *Escherichia coli* [17,18]. For the sampling of the desired number of cells, the correlation of OD_600_ and the cell count is microorganism-specific and would require the establishment of a new correlation before using the workflow with a different microorganism. The high-throughput parallel bioreactor system enables the operation of up to 48 bioreactors. Here, we worked with a lower throughput. It would be possible to increase the throughput further using additional barcoded primers.

The automated RNA-Seq sample preparation workflow saves time and reduces batch effects in library preparation. However, it comes with increased costs due to increasing reagent volumes for trueness and precision. Additionally, placing the reagents on the deck of the liquid handler requires preparing the reagents in excess, thus, further increasing costs. The high-throughput nature of the described protocols is achieved at the expense of reduced quality control. In this work, we only used the purity ratios of the nucleic acid samples to estimate RNA quality, in contrast to common practice in RNA-Seq experiments to control the quality of input RNA using RNA integrity numbers acquired by bioanalyzer measurements [61].

## Data Availability

Sequencing data have been deposited in the Sequence Read Archive (SRA) (https://www.ncbi.nlm.nih.gov/sra/?term=PRJNA1231053, accessed on 3 April 2025) with the accession number PRJNA1231053. All other data presented in this work are available upon request from the corresponding author.

## Supplementary Materials

The following supporting information can be downloaded at https://www.mdpi.com/article/10.3390/microorganisms13040849/s1, Figure S1: DO measurements of *S. cerevisiae* cultures during batch cultivation in the bioreactor unit; Figure S2: pH measurements of *S. cerevisiae* cultures during batch cultivation in the bioreactor unit; Figure S3: OD_600_, DO, pH, glucose, ethanol, and acetate concentrations (determined by HPLC measurements) of pH-controlled aerobic batch cultivations of *S. cerevisiae*; Figure S4: Quality of reads compared to length for all reads; Figure S5: Read length distributions for all libraries. Figure S6: Custom, 3D-printed lid for the Take3 Trio microvolume plate; Table S1: RNA-Seq sample annotations; Table S2: Liquid class development for SQK-PCB114.24 kit (Oxford Nanopore Technologies, Oxford, United Kingdom); Table S3: Liquid class development for Mag-Bind Total RNA 96 Kit (Omega Bio-Tek, Inc., Norcross, USA).

## References

[1] Hemmerich J., Noack S., Wiechert W., Oldiges M. (2018). Microbioreactor Systems for Accelerated Bioprocess Development. Biotechnol. J..

[2] Von den Eichen N., Bromig L., Sidarava V., Marienberg H., Weuster-Botz D. (2021). Automated Multi-Scale Cascade of Parallel Stirred-Tank Bioreactors for Fast Protein Expression Studies. J. Biotechnol..

[3] Puskeiler R., Kaufmann K., Weuster-Botz D. (2005). Development, Parallelization, and Automation of a Gas-Inducing Milliliter-Scale Bioreactor for High-Throughput Bioprocess Design (HTBD). Biotechnol. Bioeng..

[4] Kusterer A., Krause C., Kaufmann K., Arnold M., Weuster-Botz D. (2008). Fully Automated Single-Use Stirred-Tank Bioreactors for Parallel Microbial Cultivations. Bioprocess. Biosyst. Eng..

[5] Schmideder A., Hensler S., Lang M., Stratmann A., Giesecke U., Weuster-Botz D. (2016). High-Cell-Density Cultivation and Recombinant Protein Production with *Komagataella pastoris* in Stirred-Tank Bioreactors from Milliliter to Cubic Meter Scale. Process Biochem..

[6] von den Eichen N., Osthege M., Dolle M., Bromig L., Wiechert W., Oldiges M., Weuster-Botz D. (2022). Control of Parallelized Bioreactors II: Probabilistic Quantification of Carboxylic Acid Reductase Activity for Bioprocess Optimization. Bioprocess. Biosyst. Eng..

[7] Janzen N.H., Schmidt M., Krause C., Weuster-Botz D. (2015). Evaluation of Fluorimetric pH Sensors for Bioprocess Monitoring at Low pH. Bioprocess. Biosyst. Eng..

[8] Haby B., Hans S., Anane E., Sawatzki A., Krausch N., Neubauer P., Bournazou M.N.C. (2019). Integrated Robotic Mini Bioreactor Platform for Automated, Parallel Microbial Cultivation with Online Data Handling and Process Control. SLAS Technol..

[9] Benner P., Effenberger S., Franzgrote L., Kurzrock-Wolf T., Kress K., Weuster-Botz D. (2020). Contact-Free Infrared OD Measurement for Online Monitoring of Parallel Stirred-Tank Bioreactors up to High Cell Densities. Biochem. Eng. J..

[10] Wang Z., Gerstein M., Snyder M. (2009). RNA-Seq: A Revolutionary Tool for Transcriptomics. Nat. Rev. Genet..

[11] Park S.T., Kim J. (2016). Trends in Next-Generation Sequencing and a New Era for Whole Genome Sequencing. Int. Neurourol. J..

[12] Levy S.E., Myers R.M. (2016). Advancements in Next-Generation Sequencing. Annu. Rev. Genom. Hum. Genet..

[13] Hör J., Gorski S.A., Vogel J. (2018). Bacterial RNA Biology on a Genome Scale. Mol. Cell.

[14] Stark R., Grzelak M., Hadfield J. (2019). RNA Sequencing: The Teenage Years. Nat. Rev. Genet..

[15] Jain M., Olsen H.E., Paten B., Akeson M. (2016). The Oxford Nanopore Minion: Delivery of Nanopore Sequencing to the Genomics Community. Genome Biol..

[16] Jenjaroenpun P., Wongsurawat T., Pereira R., Patumcharoenpol P., Ussery D.W., Nielsen J., Nookaew I. (2018). Complete Genomic and Transcriptional Landscape Analysis Using Third-Generation Sequencing: A Case Study of *Saccharomyces cerevisiae* CEN.PK113-7D. Nucleic Acids Res..

[17] Grünberger F., Ferreira-Cerca S., Grohmann D. (2022). Nanopore Sequencing of RNA and cDNA Molecules in *Escherichia coli*. RNA.

[18] Rodger G., Lipworth S., Barrett L., Oakley S., Crook D.W., Eyre D.W., Stoesser N. (2024). Comparison of Direct cDNA and PCR-cDNA Nanopore Sequencing of RNA from *Escherichia coli* Isolates. Microb. Genom..

[19] Parapouli M., Vasileiadis A., Afendra A.S., Hatziloukas E. (2020). *Saccharomyces cerevisiae* and Its Industrial Applications. AIMS Microbiol..

[20] Mohd Azhar S.H., Abdulla R., Jambo S.A., Marbawi H., Gansau J.A., Faik A.A.M., Rodrigues K.F. (2017). Yeasts in Sustainable Bioethanol Production: A Review. Biochem. Biophys. Rep..

[21] Nielsen J. (2013). Production of Biopharmaceutical Proteins by Yeast. Bioengineered.

[22] Vieira Gomes A.M., Carmo T.S., Carvalho L.S., Bahia F.M., Parachin N.S. (2018). Comparison of Yeasts as Hosts for Recombinant Protein Production. Microorganisms.

[23] Baptista S.L., Costa C.E., Cunha J.T., Soares P.O., Domingues L. (2021). Metabolic Engineering of *Saccharomyces cerevisiae* for the Production of Top Value Chemicals from Biorefinery Carbohydrates. Biotechnol. Adv..

[24] Bozell J.J., Petersen G.R. (2010). Technology Development for the Production of Biobased Products from Biorefinery Carbohydrates—The US Department of Energy’s “Top 10” Revisited. Green. Chem..

[25] Overkamp K.M., Bakker B.M., Kötter P., Luttik M.A., Van Dijken J.P., Pronk J.T. (2002). Metabolic Engineering of Glycerol Production in *Saccharomyces cerevisiae*. Appl. Env. Microbiol..

[26] Santos L.O., Silva P.G.P., Lemos Junior W.J.F., de Oliveira V.S., Anschau A. (2022). Glutathione Production by *Saccharomyces cerevisiae*: Current State and Perspectives. Appl. Microbiol. Biotechnol..

[27] Wang Z., Zhang R., Yang Q., Zhang J., Zhao Y., Zheng Y., Yang J., Gadd G.M., Sariaslani S. (2021). Chapter One-Recent Advances in the Biosynthesis of Isoprenoids in Engineered *Saccharomyces cerevisiae*. Advances in Applied Microbiology.

[28] Gancedo J.M. (1998). Yeast Carbon Catabolite Repression. Microbiol. Mol. Biol. Rev..

[29] Frey P.A. (1996). The Leloir Pathway: A Mechanistic Imperative for Three Enzymes to Change the Stereochemical Configuration of a Single Carbon in Galactose. FASEB J..

[30] Dynesen J., Smits H.P., Olsson L., Nielsen J. (1998). Carbon Catabolite Repression of Invertase during Batch Cultivations of *Saccharomyces cerevisiae*: The Role of Glucose, Fructose, and Mannose. Appl. Microbiol. Biotechnol..

[31] Marques W.L., Raghavendran V., Stambuk B.U., Gombert A.K. (2015). Sucrose and *Saccharomyces cerevisiae*: A Relationship Most Sweet. FEMS Yeast Res..

[32] Hortsch R., Weuster-Botz D. (2010). Power Consumption and Maximum Energy Dissipation in a Milliliter-Scale Bioreactor. Biotechnol. Prog..

[33] Bromig L., von den Eichen N., Weuster-Botz D. (2022). Control of Parallelized Bioreactors I: Dynamic Scheduling Software for Efficient Bioprocess Management in High-Throughput Systems. Bioprocess. Biosyst. Eng..

[34] Dyer S.C., Austine-Orimoloye O., Azov A.G., Barba M., Barnes I., Barrera-Enriquez V.P., Becker A., Bennett R., Beracochea M., Berry A. (2024). Ensembl 2025. Nucleic Acids Res..

[35] Li H. (2018). Minimap2: Pairwise Alignment for Nucleotide Sequences. Bioinformatics.

[36] Li H. (2021). New Strategies to Improve Minimap2 Alignment Accuracy. Bioinformatics.

[37] Patro R., Duggal G., Love M.I., Irizarry R.A., Kingsford C. (2017). Salmon Provides Fast and Bias-Aware Quantification of Transcript Expression. Nat. Methods.

[38] Li H., Handsaker B., Wysoker A., Fennell T., Ruan J., Homer N., Marth G., Abecasis G., Durbin R., Subgroup Genome Project Data Processing (2009). The Sequence Alignment/Map Format and Samtools. Bioinformatics.

[39] Shen W., Le S., Li Y., Hu F. (2016). Seqkit: A Cross-Platform and Ultrafast Toolkit for Fasta/Q File Manipulation. PLoS ONE.

[40] Soneson C., Love M.I., Robinson M.D. (2015). Differential Analyses for RNA-Seq: Transcript-Level Estimates Improve Gene-Level Inferences. F1000Research.

[41] Chen Y., Chen L., Lun A.T., Baldoni P.L., Smyth G.K. (2025). edgeR V4: Powerful Differential Analysis of Sequencing Data with Expanded Functionality and Improved Support for Small Counts and Larger Datasets. Nucleic Acids Res..

[42] Benjamini Y., Hochberg Y. (1995). Controlling the False Discovery Rate: A Practical and Powerful Approach to Multiple Testing. J. R. Statist..

[43] McCarthy D.J., Smyth G.K. (2009). Testing Significance Relative to a Fold-Change Threshold Is a Treat. Bioinformatics.

[44] Yu G., Wang L.G., Han Y., He Q.Y. (2012). Clusterprofiler: An R Package for Comparing Biological Themes among Gene Clusters. Omics.

[45] Conesa A., Madrigal P., Tarazona S., Gomez-Cabrero D., Cervera A., McPherson A., Szcześniak M.W., Gaffney D.J., Elo L.L., Zhang X. (2016). A Survey of Best Practices for RNA-Seq Data Analysis. Genome Biol..

[46] Robinson M.D., Oshlack A. (2010). A Scaling Normalization Method for Differential Expression Analysis of RNA-Seq Data. Genome Biol..

[47] Turcotte B., Liang X.B., Robert F., Soontorngun N. (2010). Transcriptional Regulation of Nonfermentable Carbon Utilization in Budding Yeast. FEMS Yeast Res..

[48] Stanley D., Bandara A., Fraser S., Chambers P.J., Stanley G.A. (2010). The Ethanol Stress Response and Ethanol Tolerance of *Saccharomyces cerevisiae*. J. Appl. Microbiol..

[49] Kato S., Yoshida M., Izawa S. (2019). Btn2 Is Involved in the Clearance of Denatured Proteins Caused by Severe Ethanol Stress in *Saccharomyces cerevisiae*. FEMS Yeast Res..

[50] De Smidt O., Du Preez J.C., Albertyn J. (2008). The Alcohol Dehydrogenases of *Saccharomyces cerevisiae*: A Comprehensive Review. FEMS Yeast Res..

[51] Zhao X.-Q., Bai F.-W. (2012). Zinc and Yeast Stress Tolerance: Micronutrient Plays a Big Role. J. Biotechnol..

[52] Tegally H., San J.E., Giandhari J., de Oliveira T. (2020). Unlocking the Efficiency of Genomics Laboratories with Robotic Liquid-Handling. BMC Genom..

[53] Kong N., Ng W., Thao K., Agulto R., Weis A., Kim K.S., Korlach J., Hickey L., Kelly L., Lappin S. (2017). Automation of PacBio SMRTbell NGS Library Preparation for Bacterial Genome Sequencing. Stand. Genom. Sci..

[54] Holland I., Davies J.A. (2020). Automation in the Life Science Research Laboratory. Front. Bioeng. Biotechnol..

[55] Annona G., Liberti A., Pollastro C., Spagnuolo A., Sordino P., De Luca P. (2024). Reaping the Benefits of Liquid Handlers for High-Throughput Gene Expression Profiling in a Marine Model Invertebrate. BMC Biotechnol..

[56] Berglund E., Saarenpää S., Jemt A., Gruselius J., Larsson L., Bergenstråhle L., Lundeberg J., Giacomello S. (2020). Automation of Spatial Transcriptomics Library Preparation to Enable Rapid and Robust Insights into Spatial Organization of Tissues. BMC Genom..

[57] Jemt A., Salmén F., Lundmark A., Mollbrink A., Fernández Navarro J., Ståhl P.L., Yucel-Lindberg T., Lundeberg J. (2016). An Automated Approach to Prepare Tissue-Derived Spatially Barcoded RNA-Sequencing Libraries. Sci. Rep..

[58] Schurch N.J., Schofield P., Gierliński M., Cole C., Sherstnev A., Singh V., Wrobel N., Gharbi K., Simpson G.G., Owen-Hughes T. (2016). How Many Biological Replicates Are Needed in an RNA-Seq Experiment and Which Differential Expression Tool Should You Use?. RNA.

[59] Ueffing K., Hug N., Niggli H., Tran T.-T.-T. (2023). Hamilton’s Magpip Technology Allows Automation of Pipetting Low Volume Reactions at High Speed and Quality. https://www.hamiltoncompany.com/automated-liquid-handling.

[60] Hadimioglu B., Stearns R., Ellson R. (2016). Moving Liquids with Sound: The Physics of Acoustic Droplet Ejection for Robust Laboratory Automation in Life Sciences. J. Lab. Autom..

[61] Schroeder A., Mueller O., Stocker S., Salowsky R., Leiber M., Gassmann M., Lightfoot S., Menzel W., Granzow M., Ragg T. (2006). The RIN: An RNA Integrity Number for Assigning Integrity Values to RNA Measurements. BMC Mol. Biol..

