# Supplementary material for: Automation of RNA-Seq Sample Preparation and Miniaturized Parallel Bioreactors Enable High-Throughput Differential Gene Expression Studies
Source: Microorganisms. 2025 Apr 8;13(4):849. doi: 10.3390/microorganisms13040849 (PMC12029635; doi:10.3390/microorganisms13040849)
Supplement: Supplementary file 1 [file microorganisms-13-00849-s001.zip › microorganisms-3542642-supplementary.pdf]

Supplementary Materials

Automation of RNA-Seq sample preparation and miniaturized parallel bioreactors enable high-throughput differential gene expression studies

Karlis Blums [1], Josha Herzog [1], Jonathan Costa [1], Lara Quirico [1], Jonas Turber [1] and Dirk Weuster-Botz [1,*]

[1] Technical University of Munich, TUM School of Engineering and Design, Chair of Biochemical Engineering, Boltzmannstraße 15, D-85748 Garching, Germany

* Correspondence: dirk.weuster-botz@tum.de; Tel.: +49.89.289.15712

1. Supplementary Figures and Tables

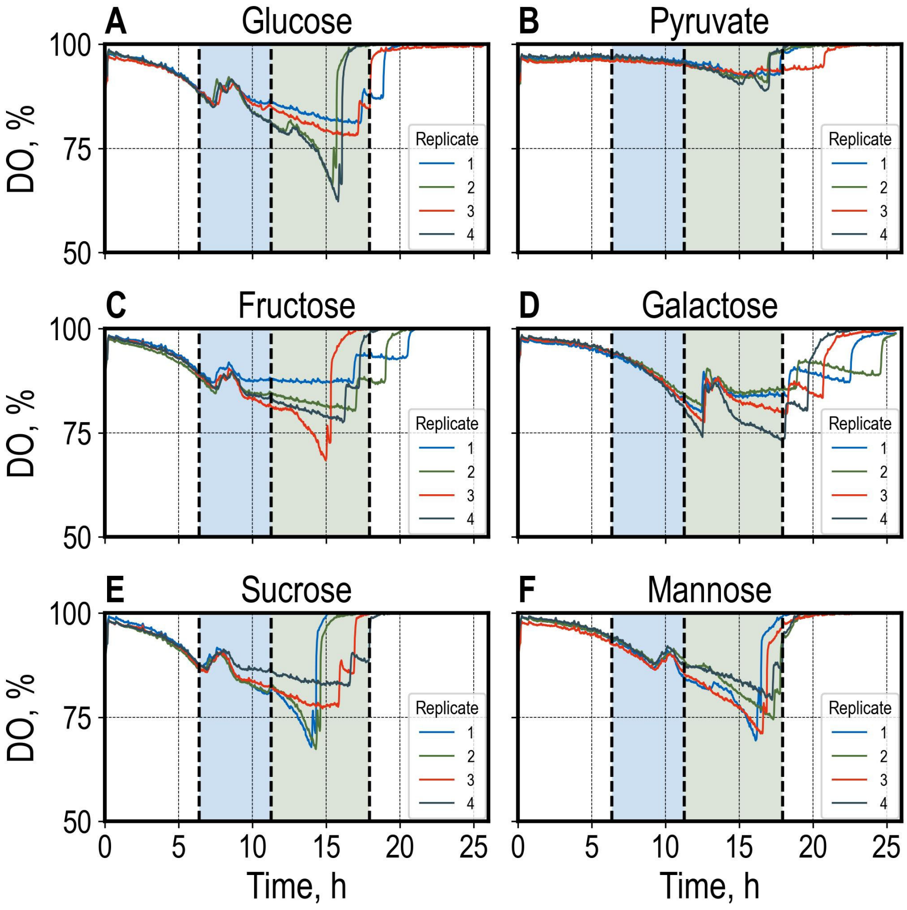

Figure S1. Dissolved oxygen (DO) measurements of *S. cerevisiae* cultures during batch cultivation in the bioreactor unit (bioREACTOR48 DS) with four biological replicates per condition. The enzymatic cell lysis and total RNA extraction, depicted by the blue area, were started for all conditions at t = 6.4 h, where the mean optical density (OD_{600}) of the glucose condition had reached $OD_{600} > 2.5$ and finished at t = 11.3 h. Library Prep was started immediately after and finished at t = 17.9 h, as shown by the green area. The following process parameters were set: V = 11 mL, gas flow rate = 0.1 L min^{-1}, stirrer speed = 3000 rpm, T = 30 °C, pH = 6.0.

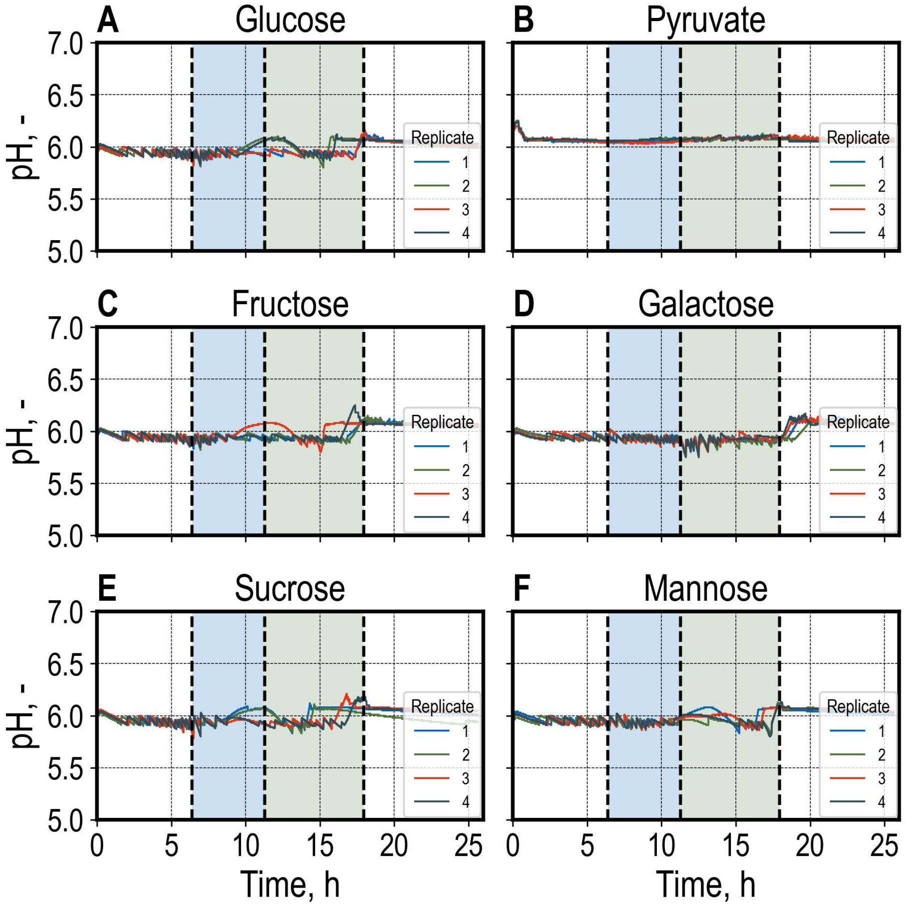

Figure S2. pH measurements of *S. cerevisiae* cultures during batch cultivation in the bioreactor unit (bioREACTOR48 DS) with four biological replicates per condition. On-line pH measurements were carried out every 40 s. The enzymatic cell lysis and total RNA extraction, depicted by the blue area, were started for all conditions at t = 6.4 h, where the mean optical density (OD_{600}) of the glucose condition had reached OD_{600} > 2.5 and finished at t = 11.3 h. Library Prep was started immediately after and finished at t = 17.9 h, as shown by the green area. The following process parameters were set: V = 11 mL, gas flow rate = 0.1 L min^{-1}, stirrer speed = 3000 rpm, T = 30 °C, pH = 6.0.

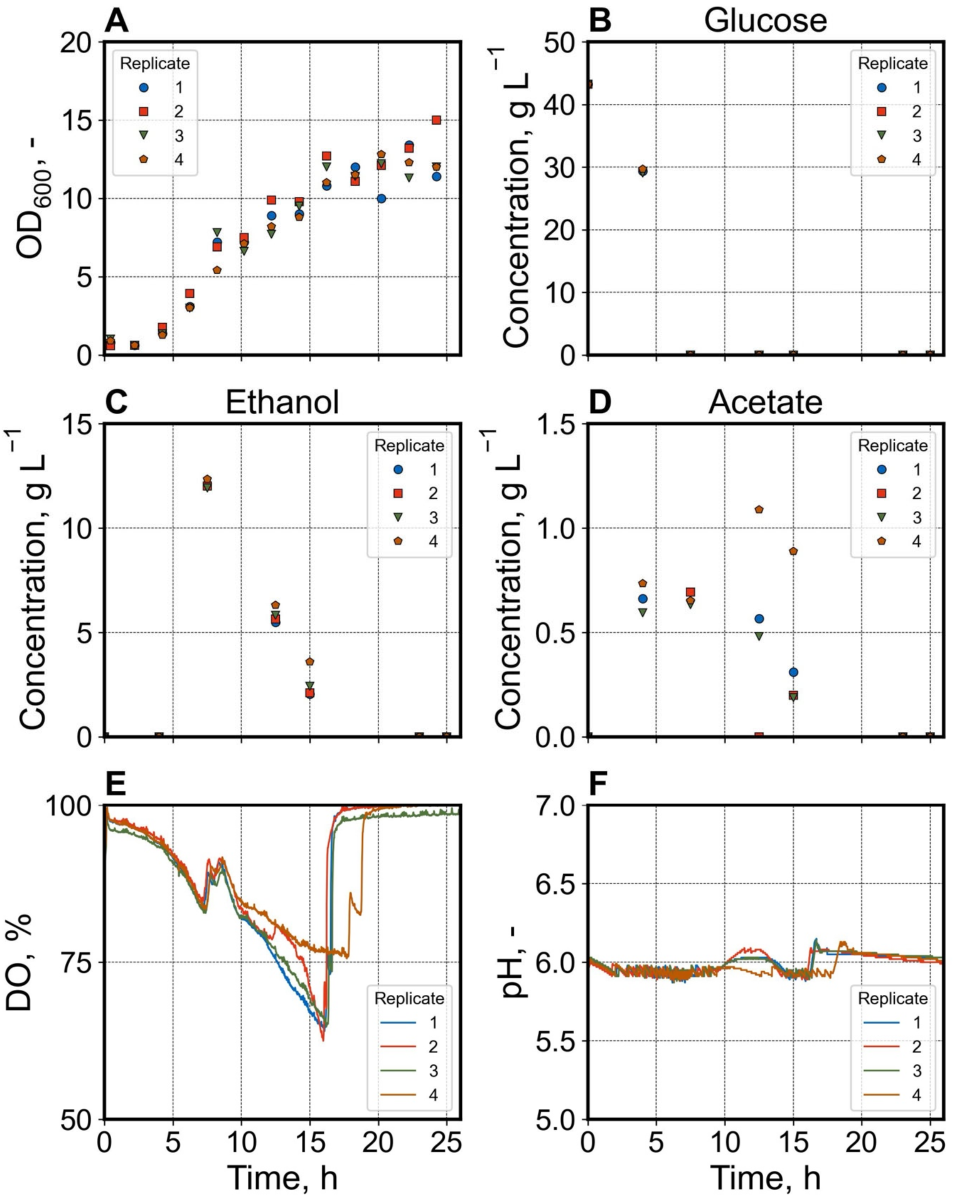

Figure S3. Optical density (OD_{600}), dissolved oxygen (DO), pH, glucose, ethanol, and acetate concentrations (determined by HPLC measurements) of pH-controlled aerobic batch cultivations of *S. cerevisiae* with 40 g L^{-1} glucose. Increased acetate consumption corresponds to a steeper pH increase in the medium and increased DO consumption. The following process parameters were set: V = 11 mL, gas flow rate = 0.1 L min^{-1}, stirrer speed = 3000 rpm, T = 30 °C, pH = 6.0. Glucose, ethanol and acetate were quantified using HPLC (1100 Series, Agilent Technologies, Santa Clara, CA, USA), which was equipped with a refractive index (RI) detector and an Aminex HPX-87H ion exchange column (Bio-Rad, Munich, Germany). The separation was achieved using 5 mM H_2SO_4 as the mobile phase at a constant flow rate of 0.7 mL min^{-1} , with the column maintained at 50 ◦C. The injection volume was 20 µL. Before injection into the HPLC system, samples were filtered through a 0.2 µm cellulose filter (Chromafil RC20/15 MS; Macherey-Nagel GmbH & Co. KG, Düren, Germany).

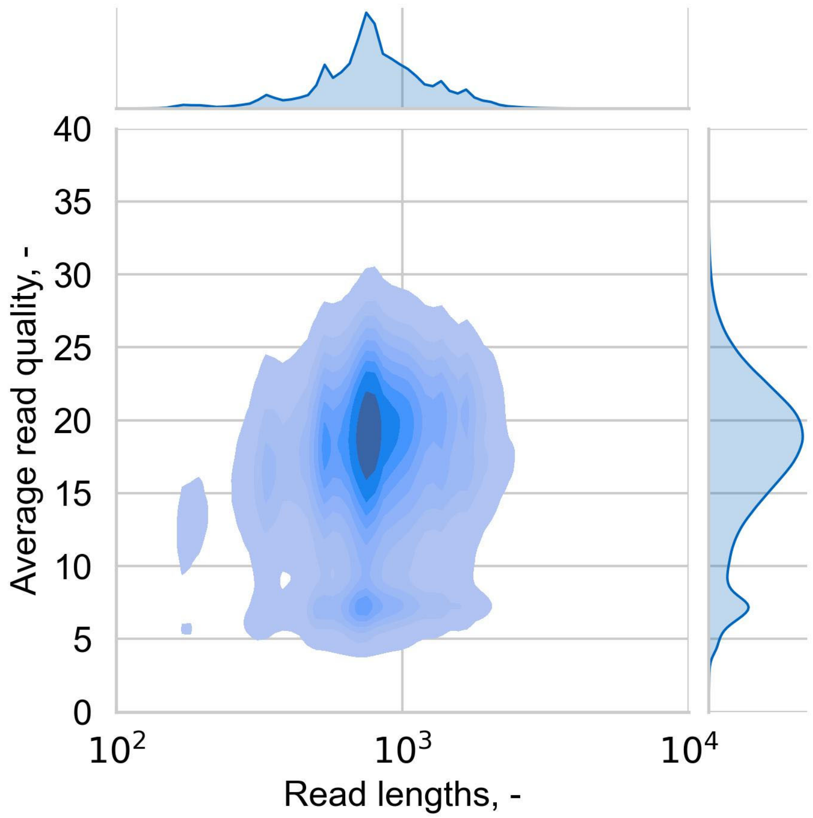

Figure S4. Quality of reads compared to length for all reads after a sequencing run on the MinION using an R10.4 flow cell over 72 h. The total yield was 24.4 M reads. The overall N50 was 918 bp, and the median read quality was 18.

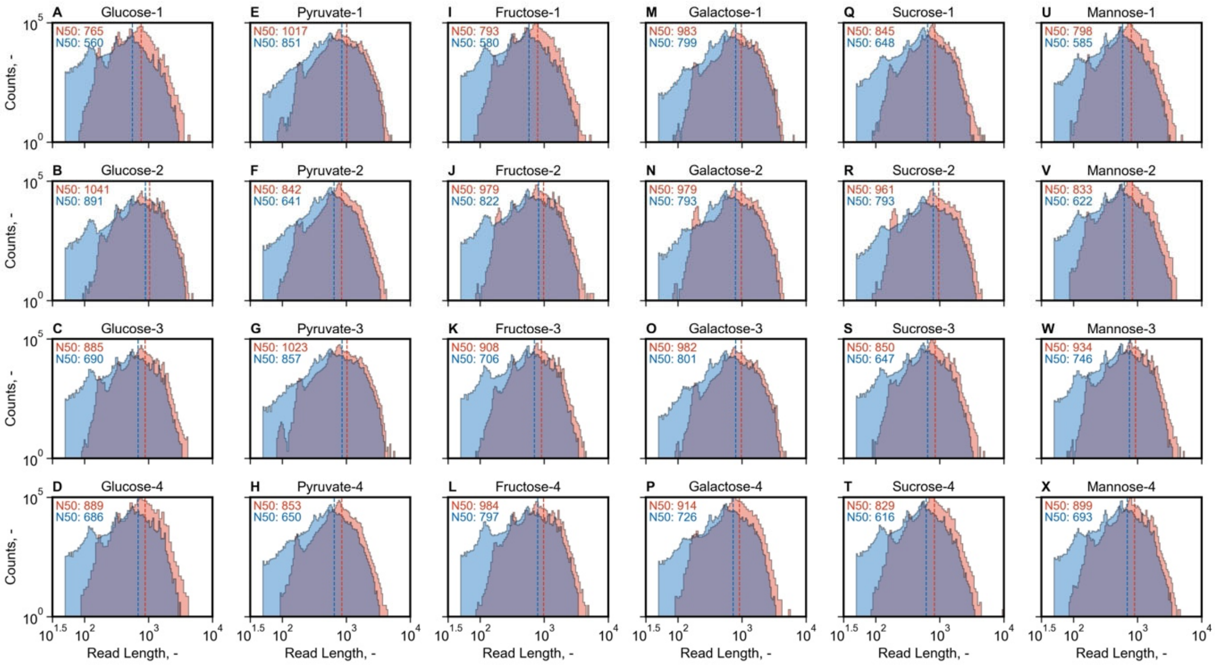

Figure S5. Read length distributions for all libraries. The raw read distributions (Q-score > 9) are shown in red, whereas the trimmed, full-length read distributions identified by Pychopper are shown in blue. Dashed vertical lines show the N50 values in bp corresponding to the color of each distribution.

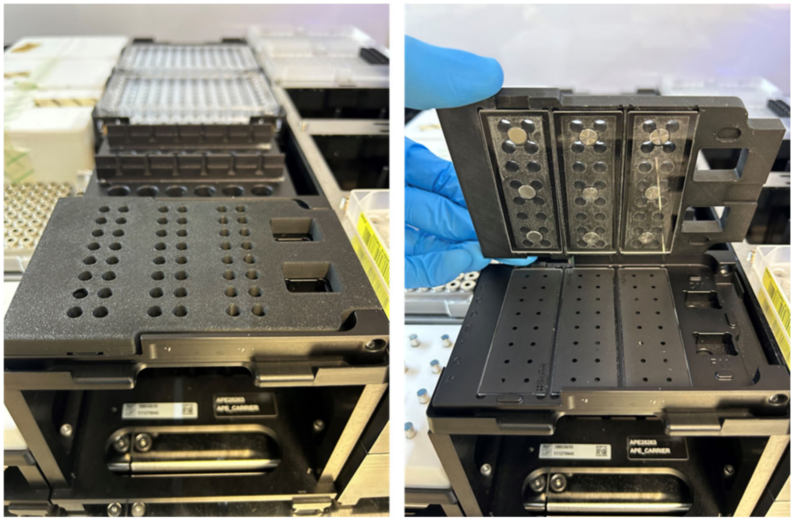

Figure S6. Custom, 3D-printed lid for the Take3 Trio microvolume plate (Agilent Technologies, Inc., Santa Clara, USA), enabling handling by the liquid handler.

Table S1. RNA-Seq sample annotations. All samples were prepared from *Saccharomyces cerevisiae* Meyen ex. Hansen bioreactor cultures, which were all sampled at a process time of 6.4 h.

Sample	Carbon source	Repli-cate	Barcode	OD_{600} at sampling time, -	Volume sampled for RNA ex., µL	RNA conc. ng μL^{-1}	A_{260}/A_{280}, -	A_{260}/A_{230}, -	cDNA conc, ng μL^{-1}	N50 of raw reads (Q>9)	N50 of full-length reads	Raw reads, -	Full-length reads, -	Primary align-ments, -	Raw bases, -	Full-length ba-ses, -	Aligned bases, -
1	Glucose	1	Barcode01	3.03	123.22	351.65	2.254	2.254	17.96	765	560	796392	652392	592090	540389598	299968458	208474243
2	Glucose	2	Barcode02	3.22	115.95	429.99	2.298	2.298	29.84	1041	891	511904	465919	449337	485071790	346221898	268005247
3	Glucose	3	Barcode03	2.30	162.33	454.60	2.24	2.24	25.08	885	690	676581	616346	579042	559292022	369619956	272533645
4	Glucose	4	Barcode04	2.70	138.28	456.96	2.286	2.286	43.39	889	686	822311	749860	708870	690684102	451154899	333381607
5	Pyruvate	1	Barcode05	0.74	504.53	110.72	2.274	2.274	12.11	1017	851	792611	725624	676937	750651706	534318349	379630188
6	Pyruvate	2	Barcode06	0.83	449.82	100.43	2.266	2.266	49.87	842	641	924020	820860	757085	768562391	496742424	328037319
7	Pyruvate	3	Barcode07	0.69	541.09	147.33	2.269	2.269	10.57	1023	857	844256	771067	719546	802748722	572218819	408007644
8	Pyruvate	4	Barcode08	0.84	444.46	103.68	2.267	2.267	26.64	853	650	839165	749379	687927	698630845	452216802	300389719
9	Fructose	1	Barcode09	2.66	140.36	293.19	2.255	2.255	37.82	793	580	1084469	941560	865020	816168863	484735706	333204704
10	Fructose	2	Barcode10	2.86	130.54	334.62	2.282	2.282	31.68	979	822	842991	742205	709329	755383319	520824063	390254747
11	Fructose	3	Barcode11	3.13	119.28	261.26	2.267	2.267	67.17	908	706	890005	810903	764362	764984577	503148603	365418675
12	Fructose	4	Barcode12	3.07	121.61	245.05	2.275	2.275	37.57	984	797	861055	796016	750492	768101622	524607634	392793492
13	Galactose	1	Barcode13	1.80	207.42	45.38	2.271	2.271	9.8	983	799	846543	785855	754213	797448069	569012786	416078698
14	Galactose	2	Barcode14	1.47	253.98	175.31	2.272	2.272	13.45	979	793	715502	640026	614314	658681064	461332002	336247370
15	Galactose	3	Barcode15	1.46	255.72	186.76	2.269	2.269	17.25	982	801	604898	561258	536942	569363847	405908191	296322819
16	Galactose	4	Barcode16	1.48	252.26	213.18	2.272	2.272	47.16	914	726	919615	846624	802777	807331342	556099966	395865006
17	Sucrose	1	Barcode17	4.0	93.34	318.09	2.275	2.275	48.51	845	648	996593	913012	853129	825386421	549231763	378691051
18	Sucrose	2	Barcode18	5.3	70.44	302.70	2.288	2.288	14.39	961	793	428845	383790	362840	382593825	268490454	192408636
19	Sucrose	3	Barcode19	4.0	93.34	330.98	2.272	2.272	53.65	850	647	1100813	990322	927898	905297424	583775946	412839842
20	Sucrose	4	Barcode20	3.7	100.91	343.75	2.278	2.278	42.49	829	616	1096628	967638	906092	885782529	550513029	384241377
21	Mannose	1	Barcode21	2.45	152.39	194.56	2.268	2.268	58.81	798	585	1035166	916031	850221	809534708	501692102	336622884
22	Mannose	2	Barcode22	2.18	171.26	220.10	2.278	2.278	69.36	833	622	1153194	988827	913668	932699752	566933482	387681739
23	Mannose	3	Barcode23	2.47	151.15	210.82	2.28	2.28	50.68	934	746	1087358	994184	932307	955869604	645644562	461358169
24	Mannose	4	Barcode24	2.21	168.94	246.45	2.28	2.28	59.5	899	693	1097789	972290	913626	944447055	607956034	428593464

Table S2. Liquid class development for SQK-PCB114.24 kit (Oxford Nanopore Technologies, Oxford, United Kingdom). Mastermix 1: cDNA RT Adapter (50% v/v) + Annealing Buffer (50% v/v), Mastermix 2: NEBNext Quick Ligation Reaction Buffer (60% v/v) + Ligase (23.3% v/v) + RNaseOUT (16.7% v/v), Mastermix 3: Lambda Exonuclease (50% v/v) + USER (50% v/v), Mastermix 4: RT Primer (50% v/v) + dNTPs (50% v/v), Mastermix 5: RT Buffer (60% v/v) + SS Primer II (26.7% v/v) + RNaseOUT (13.3% v/v), Mastermix 6: Nuclease Free Water (33.3% v/v) + Taq Master Mix (66.7% v/v). Precision is expressed in terms of the coefficient of variation (CV).

Reagent	Target Volume, µL	Trueness, %	CV, %
Mastermix 1	2	0.76	11.5
Mastermix 2	6	3.6	2.5
Mastermix 3	2	0	11.4
Mastermix 4	4	0	6.3
Mastermix 5	15	0	2.1
Mastermix 6	37.5	2.5	0.8
Barcode Primers	2	14.8	8.8
Reverse Transcriptase	2	10.4	5.8
Thermolabile Exonuclease	2	4.6	4.6
Elution Buffer	15	2.2	0.8
Short Fragment Buffer	100	1.2	0.2
70 % EtOH	100	1.1	0.63
Ampure XP Beads	36	1.9	1.3

Table S3. Liquid class development for Mag-Bind Total RNA 96 Kit (Omega Bio-Tek, Inc., Norcross, USA). Precision is expressed in terms of the coefficient of variation (CV).

Reagent	Target Volume, µL	Trueness, %	CV, %
Sorbitol Buffer	600	0	0.7
OTRK Lysis Buffer	450	0.2	0.4
RNA Wash Buffer II	300	0.7	0.8
RNA Elution Buffer	100	2.9	2.6
VHB Buffer	400	0.5	1.0
PHM Buffer	150	2.2	0.8
